# The Acari Hypothesis, VII: accounting for the comorbidity of allergy with other contemporary medical conditions, especially metabolic syndrome

**DOI:** 10.3389/falgy.2025.1537467

**Published:** 2025-03-27

**Authors:** Andrew C. Retzinger, Gregory S. Retzinger

**Affiliations:** ^1^ Department of Emergency Medicine, Camden Clark Medical Center, West Virginia University, Parkersburg, WV, United States; ^2^ Department of Pathology, Feinberg School of Medicine, Northwestern University, Chicago, IL, United States

**Keywords:** the Acari Hypothesis, eccrine glands, *Malassezia*, *Staphylococcus aureus*, metabolic syndrome, obesity, dyslipidemia, insulin resistance

## Abstract

The Acari Hypothesis proposes that vector-active acarians, i.e., mites and ticks, are the etiologic agents responsible for most, if not all, allergies. A corollary of The Hypothesis posits allergies are now more prevalent because contemporary hygienic practices remove from skin elements of sweat that otherwise deter acarians. Because the antimicrobial activity of sweat extends beyond acarians, disruption/removal of sweat on/from skin must enable aberrant microbial colonization, possibly potentiating comorbid conditions assignable to the aberrant microbial colonist(s). Allergy is strongly comorbid with metabolic syndrome. Available evidence links the principal features of metabolic syndrome to *Staphylococcus aureus*, an organism influenced significantly by constituents of sweat. Thus, the removal of sweat predisposes to both allergy and metabolic syndrome. Indeed, the “immune-compromised” state brought upon by contemporary hygienic practices likely accounts for the comorbidity of many contemporary medical conditions, examples of which are highlighted.

## 1 Introduction

Scientists and clinicians alike have long suspected that the contemporary lifestyle disrupts human physiology in ways not yet fully understood or even imagined (1). This suspicion has been prompted by the increasing incidence of contemporary medical conditions, e.g., allergy, metabolic syndrome (MetS), Alzheimer disease, polycystic ovarian syndrome, etc. (2–5). Despite disparate pathophysiologies, many contemporary conditions are comorbid with allergy (6–9), hinting that the conditions share an underlying cause. The Acari Hypothesis and its corollaries presuppose that hygienic removal of eccrine gland secretions, i.e., sweat, is responsible for the modern-day allergy epidemic (10). Removal of sweat permits aberrant acarian—human interactions, enabling generation of IgE that is deleterious.

The Acari Hypothesis was formulated to account for the clinical and epidemiological features of allergic disease (10–15). Analysis of these features, when interpreted in the context of alpha-gal hypersensitivity and synanthropic acarians, implicates dust mites, e.g., *Dermatophagoides farinae*, as causative agents of allergy (10–15).

Although The Hypothesis focuses on acarians and their involvement in allergy, a host of pathogens and their associated conditions are influenced by immune effectors found in sweat (16, 17). It stands to reason, then, that hygienic practices that disrupt and/or remove sweat must render humans vulnerable not only to acarians and allergy, but also to the other pathogens and their associated conditions. As an example, MetS, which is comorbid with asthma and atopic dermatitis (18, 19), is a contemporary disorder prevalent in developed countries (20). The symptoms of MetS, i.e., obesity, insulin resistance, hypertension (HTN) and dyslipidemia [reduced high-density lipoproteins (HDL) and elevated triglycerides and low-density lipoproteins (LDL)], have all been linked to *Staphylococcus aureus* (15, 21–24), a pathogenic gram-positive bacterium that both colonizes human skin and is sensitive to sweat (25). In keeping with The Hypothesis, disruption of sweat by hygienic practices likely renders humans vulnerable to *S. aureus* and MetS. Although The Hypothesis focuses on hygiene as the primary determinant of sweat, other factors, such as physical activity and climate control, e.g., air-conditioning, no doubt further influence the secretory activity of eccrine glands and, consequently, sweat. Not unexpectedly, the manifestations of MetS are ameliorated by regular sauna use (26–29), a measure that induces sweat. Appreciation of both the immune function of sweat and the involvement of epidermal organisms in allergy provides valuable insight into the comorbidity of allergic disease and MetS.

## 2 Discussion

### 2.1 Sweat, dermcidin, clusterin and MetS

Although a role for sweat in thermoregulation is already well-established, evidence for the involvement of sweat in innate immunity is only just developing. Five proteins constitute 94% of the protein of healthy human sweat, Table 1 (30). The two most abundant, dermcidin and clusterin, interact with the cell membrane of *S. aureus* (16, 17). Given a likely role for *S. aureus* in MetS, it is not surprising that serum levels of both dermcidin and clusterin are increased in MetS (31–33).

**Table 1:** Major proteins of eccrine gland secretions (30).

Protein	% Total protein
Dermcidin	46
Clusterin	17
Apolipoprotein D	15
Prolactin-inducible protein	8
Albumin	6

Dermcidin, the most abundant protein in sweat and the precursor of several antimicrobial peptides (30), is produced in dark cells of the secretory coils of eccrine glands. It is secreted constitutively onto epidermis (34). Once secreted, it is proteolyzed into cationic and anionic species that have broad antimicrobial activity, including activity against *Mycobacteria*, *Pseudomonas* and *Staphylococcus* (35). Anionic derivatives interact with negatively charged phospholipids of bacterial membranes, ultimately depolarizing the membranes and leading to cell death (36). Because dermcidin is the product of an orphan gene, *DCD*, unique to primates (37), there is no animal model with which to assess its loss-of-function. However, a recent family study reported the clinical consequences of dermcidin loss-of-function owing to mutated *DCD* (38). Affected family members experienced both staphylococcal overgrowth and recalcitrant hidradenitis suppurativa (HS) (38). HS is a dermatopathology linked to demodicosis. It is characterized by elevated levels of IgE, the antibody claimed central to human anti-acarian defense (39, 40). Importantly, dermcidin isoforms are elevated in sera from patients with insulin resistance and HTN (32, 33, 41), cardinal features of MetS. Such elevation is also linked to the formation of atheromatous lesions (42). Based on all of these, a role for *S. aureus* in MetS is likely.

Clusterin, or apolipoprotein J (apo J), is the second most abundant protein in the sweat of healthy individuals (30). This well-conserved mammalian glycoprotein is expressed ubiquitously in most tissues, and it is secreted abundantly in many biofluids (43, 44). It selectively binds to, and aggregates, pathogenic strains of *S. aureus* (17). This should come as no surprise because clusterin, like dermcidin, is secreted onto epidermis, the most obvious site of first contact with staphylococcal species. Clusterin has also been linked to MetS: firstly, the serum level of clusterin is elevated in persons who have insulin resistance (45); secondly, adipocytes of obese persons overexpress clusterin (46); and thirdly, advanced atheromatous lesions responsible for MetS-associated vascular disease have an abundance of platelet-derived clusterin (47). Inasmuch as clusterin likely serves an anti-staphylococcal function, it is entirely reasonable to suppose its increased expression in the serum and atheromatous lesions of persons with MetS occurs in response to increased staphylococcal burden. Further, at least some of that burden could relate to hygiene-enabled colonization of *S. aureus* on epithelial surfaces.

### 2.2 *S. aureus* and the symptoms of MetS

Individuals with MetS are at high risk for cardiovascular disease (48). The condition has been reviewed extensively by others and will not be reviewed here except to note that, like allergy, MetS was only first described in the post-hygiene era (49). Although others have linked *S. aureus* to isolated individual symptoms of MetS (50), none before has either related *S. aureus* to the condition directly or implicated a role for hygiene in the increasing incidence/prevalence of MetS.

#### 2.2.1 Obesity

Obesity has long been considered an immune-compromised state (51, 52). It confers an increased risk both for carriage of pathogenic staphylococci (53, 54) and for soft tissue infection (55, 56). Importantly, because the number of eccrine glands is fixed in early childhood (57), obesity necessarily decreases the surface density of eccrine glands, rendering it suboptimal.

As shown in Table 2, doubling the ideal body weight of an individual of average height, 175 cm, results in an estimated 60% increase in body surface area (BSA) (58). All else being equal, because over a lifetime the number of eccrine glands remains constant, the increase in BSA dilutes the two-dimensional concentration of immune effectors on the skin. Given such dilution, one might expect obese individuals to be especially vulnerable to any immune-compromising influence of hygiene, e.g., removal of sweat. There is evidence, however, that adipose tissue compensates by means of intrinsic immune activity. In mice, adipocyte proliferation and hypertrophy occur locally in direct response to epidermal inoculation of *S. aureus* (23). Adipocytes participate in anti-staphylococcal immunity via upregulation of the antimicrobial peptide, cathelicidin. They also upregulate production of clusterin (46). It is entirely plausible, then, that increased epithelial colonization by *S. aureus* promotes in humans local adipogenesis.

**Table 2:** BSA estimations by weight (58).

Weight (kg)	BSA (m^2^)
60	1.66
70	1.83
80	1.99
90	2.15
100	2.30
110	2.45
120	2.59
130	2.73
140	2.86
150	2.99

MetS is associated with visceral obesity, i.e., the disproportionate expansion of adipose tissue around the intra-abdominal organs (59). This expansion predicts risk of cardiovascular disease (60). If adipocyte proliferation and hypertrophy are consequences of local immune reactivity to *S. aureus*, then the relevant site of bacterial colonization may be intestinal epithelium, not skin. Indeed, much available evidence indicates the microbiomes of the skin and gut are interconnected/interdependent such that dysbiosis of one affects the operation of the other (61). Thus, aberrant colonization of skin by *S. aureus* may facilitate aberrant colonization of intestinal epithelium by the same organism. Relatedly, multiple studies have identified *S. aureus* in patient stools, confirming that the intestinal epithelium of at least some individuals harbors *S. aureus* routinely (62–64).

#### 2.2.2 Insulin resistance

Insulin resistance is a consequence of a dysregulated insulin responsiveness. Such dysregulation may occur following disruption of any of several molecular pathways (65, 66). Insulin resistance affects primarily liver, muscle and fat, and raises glucose levels in blood and other body fluids, including ones secreted onto epithelial surfaces, e.g., sweat, tears, saliva and airway surface liquid (ASL) (67–70). Elevated surface glucose markedly influences the sterility of the epithelial interface (71). For example, elevated ASL glucose promotes *S. aureus* colonization of human airways (72). Indeed, *S. aureus* has an expansive repertoire of glucose transporter genes that allow the organism to thrive in glucose-rich environments (73). Those genes also enable *S. aureus* to flourish under the respiration-limiting conditions effected by the human immune system. Not only is *S. aureus* specially adapted to exist in a glucose-rich environment, but the bacterium can also induce a glucose-rich environment via generation of an insulin-resistant state. In mammals, this occurs by either of two means. According to one, the extracellular enzymatic domain of lipoteichoic acid synthase binds to insulin and blocks insulin-mediated glucose uptake (22). According to the other, glucose intolerance is prompted by chronic exposure to the *S. aureus* superantigen, toxic shock syndrome toxin-1 (TSST-1) (21). In summary, *S. aureus* can manipulate a mammalian host to induce an insulin-resistant state. That state then promotes an epithelial microenvironment supportive of *S. aureus* colonization. Inasmuch as insulin resistance may prove detrimental to a mammalian host, it is entirely reasonable to suppose mammals vulnerable to such resistance would have evolved means, e.g., sweat, to deter epithelial colonization by *S. aureus*.

#### 2.2.3 HTN

HTN is defined by persistently elevated arterial blood pressure. Although the pathogenesis of HTN is still uncertain, much progress has been made in this regard. The renin—angiotensin system (RAS) appears paramount (74, 75). Briefly, renin from the kidney converts circulating liver-derived angiotensinogen to angiotensin I. Angiotensin-converting enzyme 1 (ACE1) then converts angiotensin I to angiotensin II. Subsequently, and by means of various biochemical pathways, angiotensin II increases arterial blood pressure. As evidenced by the ability of ACE1 inhibitors to abate HTN and prevent associated end-organ damage, dysregulation of the RAS appears responsible, at least in part, for MetS-associated HTN (76, 77).

To date, investigation of ACE1 expression has focused primarily on pulmonary and renal systems. ACE1, however, is also expressed abundantly both on cells lining blood vessels and on cells lining small intestine (78), the organ about which adipose tissue accumulates in persons with MetS. If the visceral obesity of MetS is a consequence of small intestinal colonization by *S. aureus*, then overexpression of ACE1 in the small intestine is also likely responsible for MetS-associated HTN.

Recent studies support a role for the RAS in innate immunity (24, 79, 80). In mice, expression of ACE1 on neutrophil membranes is upregulated following exposure of the cells to *S. aureus* (24). Overexpression of ACE1 enhances production of reactive oxygen species (ROS), thereby increasing both neutrophil bactericidal capability and survivability following bacterial phagocytosis (24). In comparison, neutrophils of either ACE1 knockout mice or mice treated with an ACE inhibitor have increased susceptibility to lysis by *S. aureus* (80). Not only neutrophils, but also ACE1-expressing enterocytes phagocytize pathogens (81–83). Although *S. aureus* has traditionally been considered an extracellular pathogen, accumulating evidence indicates the bacterium is a facultative intracellular one (84, 85). As examples, *S. aureus* exists intracellularly, both in individuals with recurrent rhinosinusitis and in individuals who are asymptomatic (86, 87). *S. aureus* can escape the phagosome of both professional and non-professional phagocytes, and it replicates intracellularly, remaining viable for up to 120 h (83). If hygiene increases exposure to, and phagocytosis of, *S. aureus* by ACE1-expressing enterocytes, then it seems likely upregulation of ACE1 by enterocytes in response to *S. aureus* contributes to systemic HTN.

Colonization of the intestine by *S. aureus* may be responsible for the association of dietary red meat with HTN, insulin resistance and heart disease (88–90). Although *S. aureus* generates fatty acids *de novo*, the bacterium routinely utilizes exogenous fatty acids, incorporating them into its membrane (91). Those fatty acids include scavenged ones from the host environment, especially during times of host infection (92, 93). Fatty acid content is a determinant of membrane fluidity (94), which, in turn, influences bacterial virulence. Evidence indicates that the SaeRS system, which controls the genes responsible for bacterial virulence, including TSST-1, is regulated by long branched-chain fatty acids (95, 96). In the modern diet, the primary sources of those fatty acids are red meat and dairy (97). From the foregoing, it stands to reason that a diet rich in red meat should predispose persons with intestinal *S. aureus* colonization to the symptoms of MetS.

#### 2.2.4 Dyslipidemia

Dyslipidemia is characterized by abnormal serum lipids, including diminished HDL and elevated triglycerides and LDL (48). Interestingly, individuals with seborrheic dermatitis (SD), a dermatopathology comorbid with MetS (98, 99), have the same lipid profile (100). Not only is SD comorbid with the dyslipidemia of MetS, but also its severity correlates positively with the degree of dyslipidemia (100). Clinical data also links SD to the events of acute coronary syndrome (101).

Although the pathophysiology of SD is still only poorly understood, epidermal *Malassezia* is believed essential to the condition. This is evidenced both by the presence of the fungus on affected skin and by the therapeutic response of SD to anti-fungal therapy (102, 103). *Malassezia* are lipid-dependent fungi that reside on the epidermis of most sebum-producing mammals (104). According to The Hypothesis and its corollaries, mutualism exists between *Malassezia* and mammals, with mammals having evolved sebaceous glands that enable malassezial colonization (15). In short, mammals provide *Malassezia* with lipid-rich secretions whilst the fungus protects mammals from parasitic acarians.

If colonization of epidermis by *Malassezia* is essential to mammalian health, then mammals likely evolved means to support the fungus during times of environmental stress. An obvious means of such support would be to increase sebum output. This could involve trafficking lipids—the raw materials of sebum—to epidermal surfaces. Consistent with this idea, sebaceous gland output is increased in persons with SD (105). As to the environmental stress eliciting such response, the epidermis of persons with SD is characterized by bacterial dysbiosis, with *S. aureus* predominating (106). Access to sebum benefits that bacterium, as evidenced by clustering of *S. aureus* around pilosebaceous units preferentially, and by colonization of *S. aureus* within the units themselves (107, 108). Like *Malassezia*, *S. aureus* produces lipases that digest sebum (109, 110). Given the nutritive requirement of *Malassezia* and the epidermal localization of *S. aureus*, the two organisms undoubtedly compete for sebum lipids. Should colonization of skin by *S. aureus* take hold by, for instance, the hygienic removal of sweat, the epidermal balance between the bacterium and fungus would favor the bacterium. Put simply, SD ensues from a specific derangement of the epidermal microbiome. Pathophysiological aspects of SD substantiate this derangement. For example, whilst the skin of SD patients reacts strongly to oleic acid, a fatty acid liberated from sebum triglycerides by malassezial lipases (111, 112), the skin of healthy persons does not. Importantly, oleic acid is deleterious to *S. aureus*: it disrupts the bacterial membrane, inhibits generation of biofilms and down-regulates virulence factors (113–115). This raises the possibility that the reactivity of the skin of persons with SD is not directed against oleic acid, but is instead directed against *S. aureus*-derived molecules expressed in response to an oleic acid challenge.

### 2.3 Atherosclerosis and myocardial infarction

Clinical recognition of MetS is important because it enables early identification of persons at increased risk of atherosclerosis, the leading cause of death in developed countries (116–118). Much about atherosclerosis has already been elaborated. Lipoproteins, including chylomicrons, chylomicron remnants, LDL and very low-density lipoprotein (VLDL) are central to the disease process (119). Briefly, relevant lipoproteins generated in cells of the intestine and/or liver traffic within the arterial circulation where they nucleate and, subsequently, model plaques beneath the intima of larger arteries. Plaque lipids activate endothelial cells, which then recruit platelets and monocytes (120–123). Activated platelets release granules containing a variety of chemical mediators, many of which are pro-inflammatory (124). Plaque-associated monocytes differentiate into lipid-laden macrophages, i.e., foam cells (125, 126). Overaccumulation of lipids within foam cells leads to apoptosis and inflammation, and to plaque necrosis, instability and, ultimately, rupture (127). Ruptured plaques, which are exceedingly thrombogenic, impede blood flow and render tissues distal to them ischemic (128).

Plaque formation warrants further consideration. Oxidized lipoproteins are consumed by macrophages (129, 130). The site at which oxidation occurs is still open to debate (131). Chylomicrons originate within enterocytes, the epithelial cells lining the small intestine. Enterocytes are phagocytic. One strategy by which phagocytic cells manage intracellular pathogens, including *S. aureus*, is generation of ROS (132, 133). Given both the phagocytic activity of enterocytes and their membranous expression of ACE1, it seems likely those cells, when infected by *S. aureus*, generate ROS and upregulate ACE1. Lipoproteins within such enterocytes would then, by virtue of proximity, become oxidized before their release into circulation. Thus, just as the existence of *S. aureus* on intestinal epithelium accounts for the HTN and visceral adiposity of MetS, the existence of *S. aureus* within enterocytes accounts for oxidation of the lipids of MetS-associated atheromatous lesions.

Because *S. aureus* associates with host lipoproteins during infection (134), those lipoproteins may retain evidence of that association. In this regard, auto-inducing peptides (AIPs) unique to *S. aureus* and secreted in response to fluctuations in bacterial population density, i.e., quorum sensing, are ideally suited (135). AIPs accumulate outside the bacterium, eventually reaching a concentration at which the bacterial accessory gene regulator (agr) becomes activated. Once activated, the agr stimulates production of staphylococcal virulence factors, including TSST-1 (136). Recent studies indicate the B apolipoproteins, constituents of chylomicrons, chylomicron remnants, LDLs and VLDLs (137), participate not only in lipoprotein formation and trafficking, but also in defense against *S. aureus* (138). In the presence of oxidized lipoprotein lipids, the B apolipoproteins bind to, and sequester, AIPs (139). Thus, at least some of the building blocks of lipoproteins oxidized in response to intracellular *S. aureus* will have AIPs bound to them. Those AIPs could then amplify inflammation triggered by oxidized plaque lipids. The presence of such a staphylococcal “marker” on the oxidized lipids would also explain the deposition of clusterin, an anti-staphylococcal agent, by platelets infiltrating the plaque (47).

### 2.4 Thiamine and peripheral neuropathy

Persons with MetS are at increased risk of peripheral neuropathy (140, 141). If *S. aureus* is an etiological agent of MetS, then the bacterium may, in fact, drive the development of neuropathy. Importantly, intestinal colonization by *S. aureus* has already been linked to peripheral neuropathy: *S. aureus* in the duodenum of a patient sequestered dietary thiamine, thereby deranging systemic thiamine metabolism which yielded, in turn, debilitating polyneuritis (142).

In mammals, thiamine serves as a critical co-factor for enzymes involved in the metabolism of lipids, carbohydrates and branched-chain amino acids. Thiamine deprivation causes neuropathy in diet-dependent fashion, i.e., beriberi (143, 144). Furthermore, in humans, mutation in the thiamine transporter, solute carrier family 19 member 2 (SLC19A2), is characterized by hyperglycemia (145). Given these, *S. aureus* may disrupt thiamine metabolism, driving hyperglycemia and ultimately producing a glucose-rich epithelial surface that benefits the bacterium. Consistent with this proposal, there are substantial bodies of evidence that thiamine supplementation protects against the development of MetS and that persons with MetS have lower serum levels of thiamine analytes (146–149).

### 2.5 Other comorbidities, including cancer

Just as allergic disease and MetS are comorbid, so, too, are other conditions comorbid with MetS, including, but not limited to, Alzheimer disease, Parkinson disease, polycystic ovary syndrome and thyroid disease (150–153). Although beyond the scope of this report, mechanistic understandings of these diseases would likely be accelerated if subject matter experts interpreted relevant data in the context of the human epithelial microbiome. In short, contemporary diseases comorbid with allergy and/or MetS undoubtedly involve acarians, *S. aureus* and/or other microbes influenced by eccrine gland secretions.

Many cancers, particularly ones derived from epithelium, e.g., adenocarcinoma of the colon, are comorbid with MetS (154). Interestingly, many of these cancers have been shown to dysregulate expression of clusterin and/or dermcidin (155–161). Such dysregulation not only renders tumor cells refractory to conventional chemo- and radiation therapies, but it also confers upon them metastatic potential (162, 163). Inasmuch as MetS is attributable to hygienic disruption of sweat and consequent epithelial colonization by *S. aureus*, it seems possible dysregulated clusterin and dermcidin in malignant epithelial cells is due to what the cells perceive as an unremitting staphylococcal challenge.

At the cellular level, clusterin plays an important role in stress responses and survival (164, 165). It has two isoforms, an intranuclear one and a secreted one, the latter of which operates both within the cytoplasm and extracellularly (166). Intranuclear clusterin is pro-apoptotic whilst the secreted isoform is anti-apoptotic (167). Secretory clusterin is an ATP-independent chaperone with very broad substrate specificity (165). As a chaperone, it is unusual in that it operates extracellularly (168). Because secretory clusterin regulates apoptosis, it seems intuitive that its dysregulation would yield a pro-oncotic state. That being the case, it is tempting to speculate that elimination of epithelial pathogens that dysregulate clusterin might not only prevent malignant transformation but might also provide therapy for some cancers.

## 3 Closing

Per The Hypothesis and its corollaries, many diseases of modern man are attributable to disruption of the human microbiome by direct and/or indirect means. As suggested above, this new understanding provides rationale for novel approaches to disease treatment. As examples, some diseases might be ameliorated either by limiting certain hygienic practices, e.g., showering daily, or by applying topical agents that mimic endogenous immune effectors, e.g., dermcidin. To better appreciate the full disease-related impact of hygiene, comparison should be made of the microbes colonizing epithelial surfaces of persons from industrialized societies with those colonizing epithelial surfaces of persons from primitive societies. Microbes colonizing the former but not the latter should be considered potential agents of modern disease(s).

The symptomatology and pathology of allergy are attributable to specious IgE-mediated adaptive responses. In persons not yet allergic, eliminating some hygienic practices should help prevent the development of allergy. Because synanthropic mites likely elicit most allergies, an evaluation of the environment of allergic persons should be undertaken to identify the causative acarian. In this regard, nucleic acid-based testing might prove useful, as mite infestation is ofttimes not apparent clinically.

Treatment of MetS is more subtle and involved. Certainly, increasing sweat would be important because sweat renders human epidermis inhospitable to *S. aureus*. Unfortunately, because MetS is associated with autonomic neuropathy (169, 170), the ability of colonized individuals to sweat may be compromised. Furthermore, bacterial colonization can extend beyond epidermis, to involve the mucosae of the airway and/or the gastrointestinal tract. More research is needed to identify immune effectors operating to deter *S. aureus* from all these surfaces.

Evidence in support of The Hypothesis might be obtained from autopsies of decedents who died from myocardial infarction and who had both clinical evidence of visceral adiposity and methicillin-resistant *Staphylococcus aureus* (MRSA) infection. Segments of the small bowel that display significant visceral adiposity could be analyzed for: (1) degree of epithelial ACE expression, and (2) presence of intracellular staphylococci. For comparative purpose, these same analyses could be performed using either less fatty segments of small intestine or segments of small intestine from healthy individuals. Although such analyses would not confirm causality, they would represent a preliminary step toward validating Koch's postulates.

Although oral and/or parenteral antibiotics could play a role in the treatment of MetS, their use might be problematic because the drugs do not discriminate between native organisms and invasive pathogens. Evidence indicates native flora protect mammals from microbial pathogens, including *S. aureus*. Commensal coagulase-negative staphylococci, e.g., *S. epidermidis*, produce bacteriocins and antimicrobial peptides (AMPs) that work synergistically with cathelicidin to inhibit epithelial colonization by *S. aureus* (171, 172). Indeed, reintroduction of coagulase-negative staphylococci onto skin of patients with atopic dermatitis limits *S. aureus* colonization (172).

Just as reintroducing coagulase-negative staphylococci onto the skin of patients with atopic dermatitis limits epidermal colonization by *S. aureus*, replenishing and/or supplementing gut microbiota with *Bacillus subtilis* limits *S. aureus* colonization of gut epithelium (173). *B. subtilis*, a gram-positive bacterium found routinely in the gastrointestinal tract of mammals, produces two lipopeptides bactericidal for *S. aureus*, surfactin and plipastatin (174). Once daily ingestion of a probiotic containing *B. subtilis* eliminates 97% of *S. aureus* from the gut and 65% of *S. aureus* from the nares (173). For these reasons, supplementation with relevant microbiota might benefit individuals with MetS.

Unfortunately, because most antibiotics do not discriminate between beneficial and pathogenic bacteria, pairing an antibiotic with hygienic disruption/removal of sweat could yield a dysbiosis that favors a pathogen. Indeed, studies have shown that antibiotic usage predisposes persons to MetS (175, 176). Because the native microbiota plays a role in human defense, its application to relevant epithelial surfaces may represent *the* most effective therapy for MetS, perhaps even a curative one.

Mammals other than humans are also subject to epithelial insult by *S. aureus* (177). That being the case, they, too, are likely to produce AMPs that selectively deter the bacterium. Indeed, epithelial secretions from mammalian sources may represent pharmaceutically useful agents. In this regard, breast milk seems especially promising: not only does it contain and support the growth of multiple microorganisms, but it also contains compounds that selectively inhibit the growth of *S. aureus* (178, 179). Furthermore, because mammalian mothers provide breast milk to their offspring, the microorganisms in that medium undoubtedly align with the intended health and fitness of the offspring.

The Acari Hypothesis provides rationale not only for allergy, but also for diseases of modernity comorbid with allergy. The authors hope this new understanding prompts serious consideration of both the epithelial microbiome and aerobiological elements in health and disease.

## Data Availability

The original contributions presented in the study are included in the article/Supplementary Material, further inquiries can be directed to the corresponding author.
